# PIAS1 Regulates Hepatitis C Virus-Induced Lipid Droplet Accumulation by Controlling Septin 9 and Microtubule Filament Assembly

**DOI:** 10.3390/pathogens10101327

**Published:** 2021-10-15

**Authors:** Abdellah Akil, Peixuan Song, Juan Peng, Claire Gondeau, Didier Samuel, Ama Gassama-Diagne

**Affiliations:** 1 Unité 1193, INSERM, F-94800 Villejuif, France; akil@uab.edu (A.A.); songpx027@gmail.com (P.S.); sammialone999@hotmail.com (J.P.); didier.samuel@aphp.fr (D.S.); 2 UMR-S 1193, Université Paris-Saclay, F-94800 Villejuif, France; 3 Department of Radiation Oncology, University of Alabama at Birmingham, Birmingham, AL 35205, USA; 4 Unité 1183, INSERM, Université Montpellier 1, F-34293 Montpellier, France; claire.gondeau@inserm.fr; 5 Department of Hepato-Gastroenterology A, Institute of Research in Biotherapy, CHU Montpellier, Hopital Saint Eloi, F-34293 Montpellier, France; 6 Centre Hépato-Biliaire, Hôpital Paul-Brousse, Assistance Publique-Hôpitaux de Paris, Université Paris-Saclay, F-94800 Villejuif, France

**Keywords:** HCV, PIAS1, septin 9, lipid droplets

## Abstract

Chronic hepatitis C virus (HCV) infection often leads to fibrosis and chronic hepatitis, then cirrhosis and ultimately hepatocellular carcinoma (HCC). The processes of the HVC life cycle involve intimate interactions between viral and host cell proteins and lipid metabolism. However, the molecules and mechanisms involved in this tripartite interaction remain poorly understood. Herein, we show that the infection of HCC-derived Huh7.5 cells with HCV promotes upregulation of the protein inhibitor of activated STAT1 (PIAS1). Reciprocally, PIAS1 regulated the expression of HCV core protein and HCV-induced LD accumulation and impaired HCV replication. Furthermore, PIAS1 controlled HCV-promoted septin 9 filament formation and microtubule polymerization. Subsequently, we found that PIAS1 interacted with septin 9 and controlled its assembly on filaments, which thus affected septin 9-induced lipid droplet accumulation. Taken together, these data reveal that PIAS1 regulates the accumulation of lipid droplets and offer a meaningful insight into how HCV interacts with host proteins.

## 1. Introduction

Hepatitis C virus (HCV) infection is a major risk factor for the development of hepatocellular carcinoma (HCC) [1]. Although new therapeutic approaches (such as direct acting antivirals; DAAs) seem to eradicate HCV, they do not completely eliminate the risk of HCC, especially in patients with advanced cirrhosis [2]. The role of hepatic steatosis in the pathogenesis of chronic hepatitis C, including fibrosis, cirrhosis, and hepatocellular carcinoma (HCC), has been shown. Although there have been several reports on the processes potentially involved, the mechanisms underlying the development of steatosis in the context of HCV infection require further investigation [3,4]. A hallmark of HCV infection is the presence of steatosis with an accumulation of lipid droplets (LDs) in the liver, which can be detected in up to 70% of infected individuals [5].

LDs are dynamic organelles that contribute to a variety of cellular functions, including the sequestration of toxic lipids [6,7], membrane biosynthesis [8,9], and lipid signaling pathways [10,11]. LDs also interact with other organelles to fulfil diverse functions and maintain cellular homeostasis [12,13,14]. We previously reported an elevated expression of septin 9 in HCV-induced cirrhosis: HCV infection increased septin 9 expression and promoted its assembly into filaments, and septin 9 regulated LD growth, dependent on microtubules (MTs). We also showed that these effects of septin 9 depend on its binding to phosphoinositides (PIs) [15].

Septins form a family of GTP-binding proteins that comprise 13 identified members in mammals [16,17]. Septins form hetero-oligomeric complexes and higher-order structures (including filaments and rings), associate with cell membranes, and organize actin and microtubule cytoskeletons [16,18,19,20,21]. Septin assembly is necessary to assure their multiple cellular functions and contribute to pathological conditions, including neurological diseases [22,23,24,25], infection by several pathogens [26,27,28,29,30,31], and cancer [32,33,34,35,36,37]. Nevertheless, while many studies have provided data on the structural organization of septins, little is known about the cellular factors responsible for septin assembly [38,39,40,41,42]. We recently identified a second polybasic PB domain in septin 9, which is conserved in all mammalian septins and is involved in their interactions with PIs and their filamentous assembly [43]. Alongside their regulation by PIs, the assembly of septins is regulated by several molecules or post-translational modifications [44] such as SUMOylation [41,42,44,45,46], which involves the covalent addition of a small ubiquitin-like modifier (SUMO) polypeptide to target proteins. In yeast, septins were among the first proteins reported as being modified by SUMOylation [46,47], a process mediated by yeast Siz proteins, which are homologous with the mammalian protein inhibitor of activated STAT1 (PIAS1) [48]. The PIAS family of proteins consists of four members: PIAS1, PIAS2 (PIASx), PIAS3, and PIASy (PIAS4), which mainly function as small ubiquitin-like modifier (SUMO) E3 ligases, regulating the function of numerous transcription factors and hence diverse cellular processes, including cell proliferation [49], DNA damage responses [50,51], and inflammation responses [52,53,54]. In mammals, the critical role of SUMO modification of the specific septins 6, 7, and 11 in septin filament formation and cell division has been reported. [42]. Nevertheless, the effects of SUMO ligases such as PIAS1 on the assembly and functions of mammalian septins have not been investigated.

In this study, we explored the potential role of PIAS1 as a regulator of septin 9 assembly and its function in HCV pathogenesis. We demonstrated the upregulation of PIAS1 expression following infection by HCV. PIAS1 colocalized with the HCV core in the perinuclear region. The knockdown of PIAS1 affected LD growth and HCV replication. PIAS1 interacted with septin 9 and regulated the formation of its filamentous structure and MT organization. Finally, we demonstrated that PIAS1 is an essential regulator of septin 9 function in HCV-infected cells. 

## 2. Result

### 2.1. HCV Infection Increases the Expression of PIAS1, Which Co-Localizes with Its Core Protein

**Figure 1:**
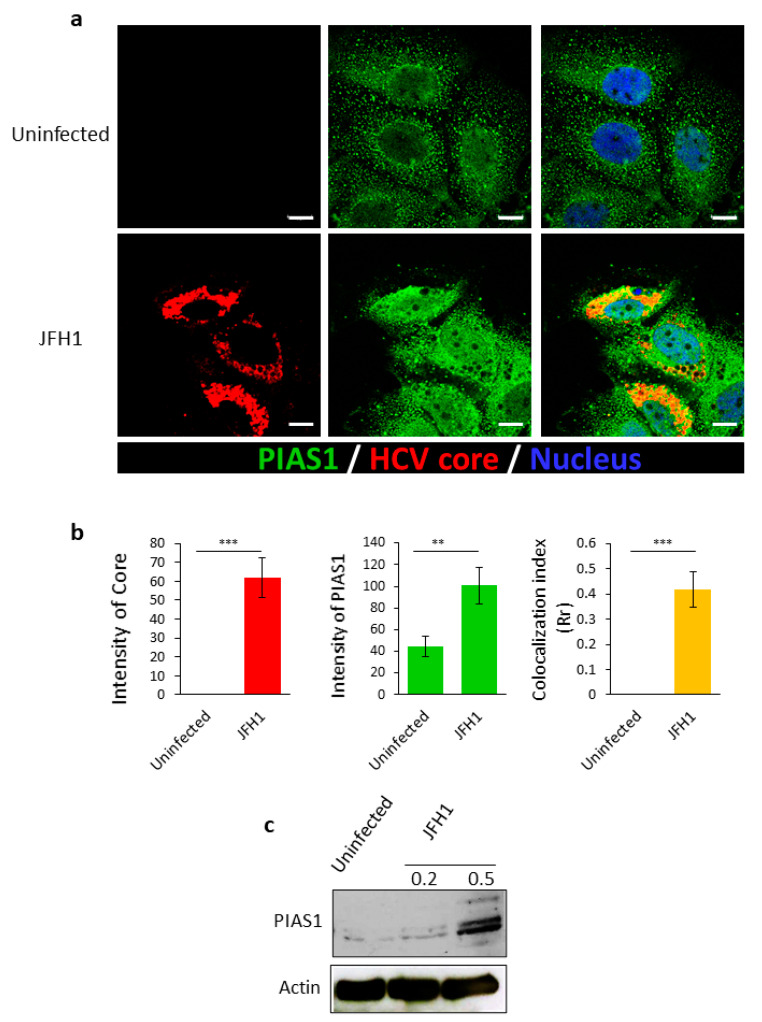
HCV infection upregulates PIAS1 expression. (**a**) Huh7.5 cells were grown overnight. The cells were then infected or not with JFH1 (0.5 Geq/cell) for 72 h, then stained for PIAS1 (green) and the HCV core (red). Scale bar, 10 µm. (**b**) The HCV core and PIAS1 intensity and the colocalization index were analyzed between the HCV core and PIAS1 of at least 30 cells from the experiments, as described in (**a**). (**c**) Huh7.5 cells were grown overnight. The cells were then infected or not with JFH1 for 0.2 or 0.5 Geq/cell for 72 h, and then analyzed by immunoblotting for PIAS1. Data information: Bar graphs present the mean ± SEM. A Student’s *t*-test was used: ** p < 0.001, and *** p < 0.0001.

**Figure 2:**
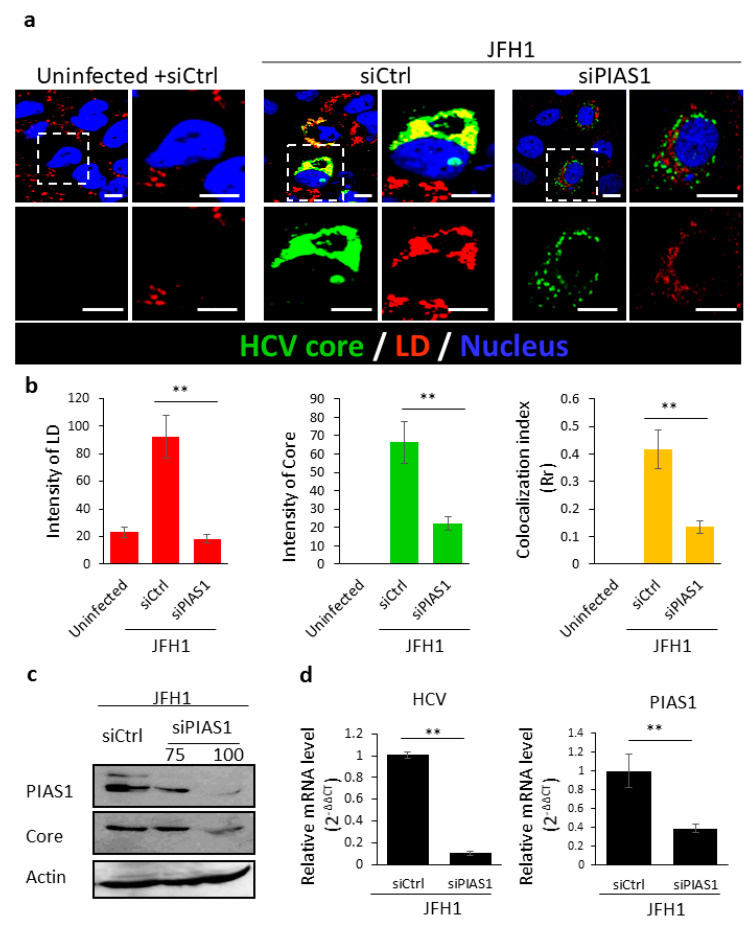
Knockdown of PIAS1 downregulates HCV replication and induces LD accumulation. (**a**) Huh7.5 cells were grown overnight. The cells were then transfected with control siRNA (siCtrl) or PIAS1 siRNA (siPIAS1) and infected or not with JFH1 for 72 h, before being stained for the HCV core (green) and LD (red), Scale bar, 10 µm. (**b**) LDs, the HCV core intensity, and the colocalization index were analyzed between the HCV core and LDs of at least 30 cells from the experiments described in (**a**). (**c**) Huh7.5 cells were grown overnight. The cells were then transfected with control siRNA (siCtrl) or PIAS1 siRNA (siPIAS1) (75 or 100 pmoles/assay), infected with JFH1 for 72 h and then analyzed by immunoblotting for PIAS1 and the HCV core. (**d**) RT-PCR assays were used to analyze PIAS1 mRNA and HCV RNA isolated from cells transfected with control siRNA (siCtrl) or PIAS1 siRNA (siPIAS1) (100 pmoles/assay) and JFH1-infected cells. Data information: Bar graphs present the mean ± SEM. A Student’s *t*-test was used: ** *p* < 0.001.

We previously reported that septin 9 plays a role in HCV pathogenesis [15] and we hypothesized that this might be mediated by its modification by PIAS1. We therefore investigated the role of PIAS1 in HCV infection, using a cell culture infection system based on Huh7.5 cells infected with the JFH-1 (Japanese fulminant hepatitis 1) strain. We inoculated Huh7.5 cells with JFH1 particles for 72 h and the cells were fixed, stained for HCV core and PIAS1, and then analyzed by confocal microscopy (Figure 1a). In non-infected cells (Uninfected), PIAS1 displayed a fine punctate distribution, while in cells infected with HCV-JFH1 (JFH1), PIAS1 expression increased and colocalized with the HCV core protein in the perinuclear region (Figure 1b), although an increase in the nuclear staining of PIAS1 was also observed (Figure 1a). We further analyzed the expression of PIAS1 by immunoblotting (Figure 1c); HCV infection increased PIAS1 expression and promoted its colocalization with the HCV core protein in the perinuclear region. PIAS1 regulates HCV-induced LD accumulation and HCV replication, and LDs play a crucial role in HCV replication and its pathogenesis [55]. Therefore, in order to assess the role of PIAS1 in the HCV life cycle, Huh7.5 cells were transfected with PIAS1-specific siRNA and then infected with JFH1 particles. As presented in Figure 2a, LDs were dispersed in the cytoplasm in the control cells (Uninfected + siCtrl), whereas in the JFH1-infected cells (JFH1 + siCtrl), there was an increase in and redistribution of LDs, as they are clustered and colocalized with the core protein in the perinuclear region (Figure 2a,b). The knockdown of PIAS1 strongly reduced the intensity of LDs and the HCV core, as well as the formation of their colocalized perinuclear clusters (Figure 2a,b). Interestingly, these findings are similar to those reported for septin 9 knockdown [15]. We also validated the protein decrease in PIAS1 and the HCV core using immunoblotting (Figure 2c). Subsequently, we examined the contribution of PIAS1 to HCV replication. Thus, Huh7.5 cells were first treated with PIAS1 siRNA and then infected with JFH1 particles, after which the total mRNA was isolated and analyzed by RT-PCR. As the results showed, the knockdown of PIAS1 induced a decrease in HCV RNA (Figure 2d), and analysis of the PIAS1 mRNA levels was used as a control for siRNA efficiency. Taken together, these different results indicate that PIAS1 contributes to the intracellular accumulation of LDs and the replication of HCV.

### 2.2. PIAS1 Regulates the HCV-Induced Stabilization of Microtubules and Endogenous Septin 9 Filaments

**Figure 3:**
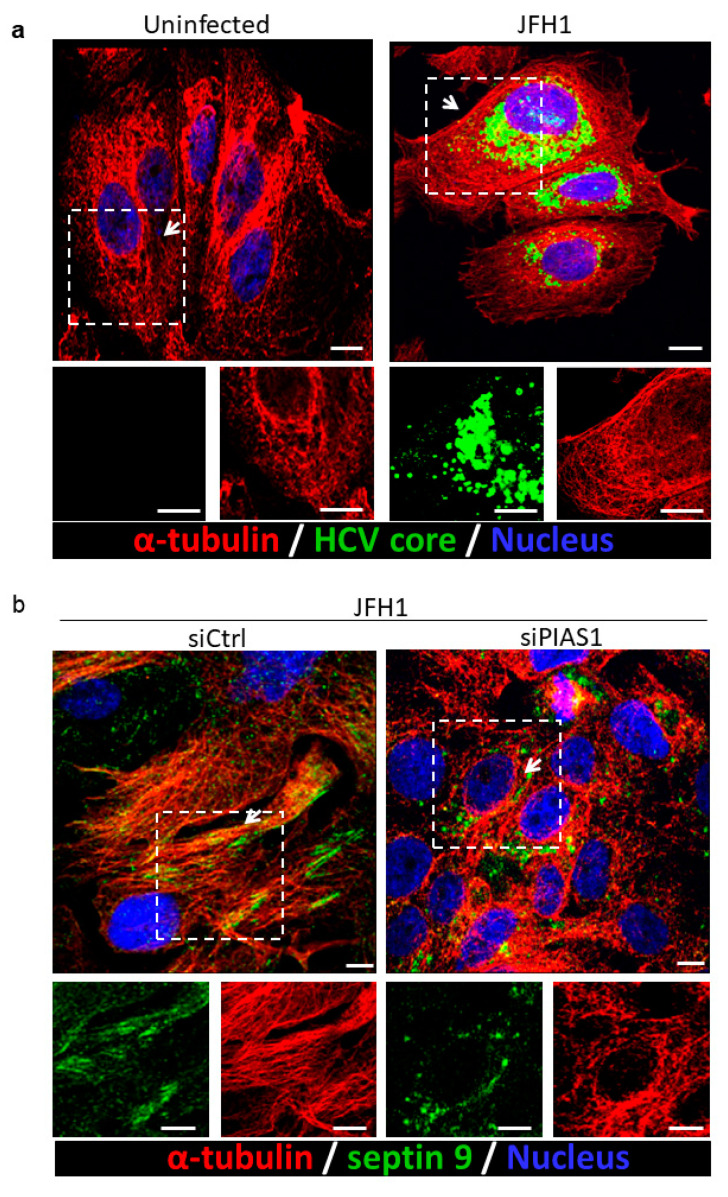
Knockdown PIAS1 disrupts HCV-induced septin 9 filament formation and MT association (**a**). Huh7.5 cells were grown overnight. The cells were then infected or not with JFH1 for 72 h and stained for the HCV core (green) and α-tubulin (red). Scale bar, 10 µm. (**b**) Huh7.5 cells were grown overnight. The cells were transfected with control siRNA or PIAS1 siRNA and infected with JFH1 for 72 h, then stained for septin 9 (green) and β-tubulin (red). Scale bar, 10 µm.

The dynamic microtubule (MT) network is critical for HCV infection [56]. Indeed, microtubules are necessary for the virus to enter cells and for particle assembly. The HCV core protein binds directly to tubulin and enhances microtubule polymerization [56]. Moreover, the redistribution of LDs induced by the HCV core protein is dependent on MTs and dynein [57], and we previously reported that septin 9 regulates the formation of MT filaments in HCV-infected cells [15]. According to these different reports and the data above, we postulated that PIAS1 might control septin 9 and MT filament formation. First, we validated the presence of the HCV core protein, which was co-stained with MTs in JFH1-infected cells (Figure 3a). We then stained septin 9 and MT filaments, which subsequently formed filaments that partly co-localized (Figure 3b), as previously reported [15]. Strikingly, the treatment of HCV-infected cells with PIAS1 siRNA abolished both MT and septin 9 filaments, and septin 9 displayed a vesicular structure instead (Figure 3b), thus indicating the critical role of PIAS1 in the formation of endogenous septin 9 and MT filaments.

### 2.3. PIAS1 Interacts with Septin 9_i1 and Controls Its Filamentous Structure Formation and MT Organization in the Presence of the HCV Genomic Replicon

**Figure 4:**
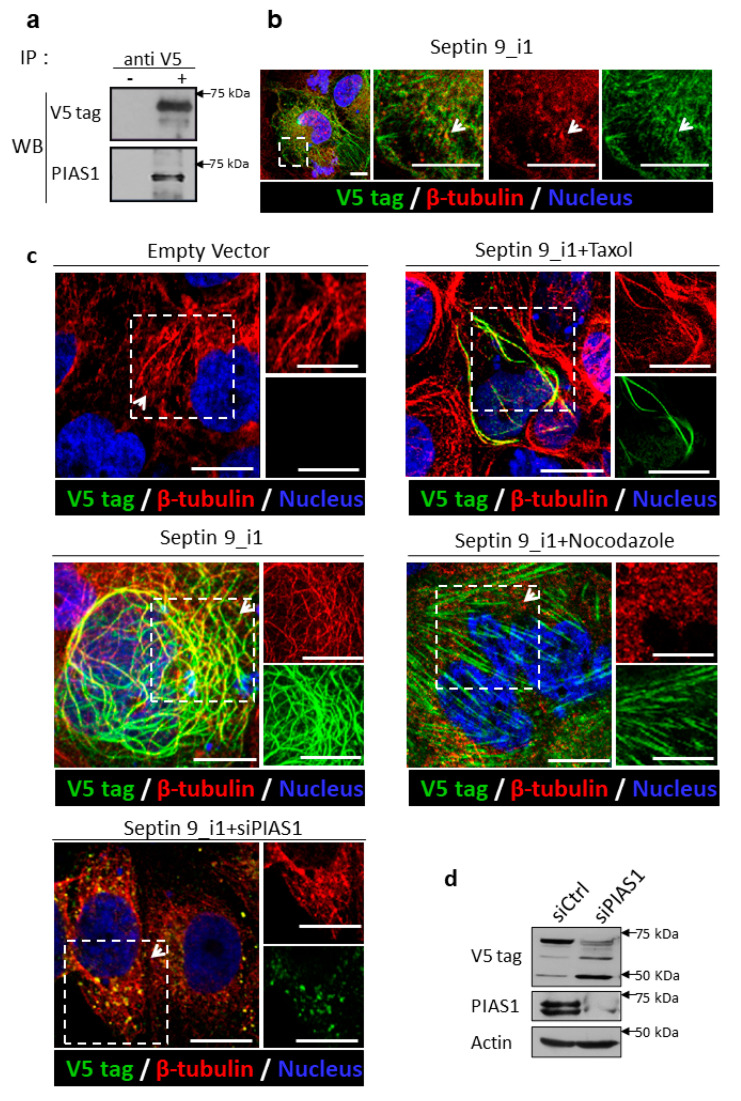
PIAS1 interacts with septin 9_v1 and affects septin 9_v1 filament formation. (**a**) Huh7R cells were transfected with septin 9_i1 and IP was performed for V5 and PIAS1. (**b**) Huh7R cells were transfected with septin 9_i1 and stained for V5 (green) and PIAS1 (red). Scale bar, 10 µm. (**c**) Huh7R cells were transfected with an empty vector or septin 9_i1 with or without PIAS1 siRNA, and stained for V5 (green) and α-tubulin (red). Scale bar, 10 µm. For the nocodazole and Taxol treatments, 33 nM nocodazole or 500 nM Taxol were added to the cells 2 h before fixation. (**d**) Huh7R cells were grown overnight. The cells were then transfected with septin 9_i1 cDNA plasmids and control siRNA (siCtrl) or PIAS1 siRNA (siPIAS1) for 48 h, and analyzed by immunoblotting for PIAS1 and the V5 tag, with actin being used as a loading control.

To confirm the data obtained with endogenous septin 9 using the JFH1 model, and to further investigate the relationship between septin 9, PIAS1, and HCV, we used Huh7 cells stably expressing the HCV genomic replicon (Huh7R) [58] to perform further mechanistic studies. First, we tried to determine probable interactions between septin 9 and PIAS1 by performing immunoprecipitation (IP). Huh7R cells were transfected with the cDNA of an empty vector (EV) and the isoform 1 of septin 9 (septin 9_i1), which has the highest expression in HCV-infected cells among the five characterized septin 9 isoforms [15]. As shown in Figure 4a, PIAS1 was revealed by immunoblotting in the immunoprecipitated of septin 9_i1 using the V5 tag antibody, thus indicating a cellular interaction between septin 9 and PIAS1 (Figure 4a). We further confirmed the presence of an endogenous PIAS1 signal associated with septin 9_i1 filament by immunofluorescence performed on cells transfected with septin 9_v1 (Figure 4b). Subsequently, we validated the effect of PIAS1 on MT and septin 9 filaments in septin 9_i1-transfected Huh7R cells. The septin 9_i1 formed clear filaments that partly colocalized with MTs (Figure 4c), and treatment with PIAS1 siRNA impaired the formation of septin 9_i1 filaments (Figure 4c). In this case, septin 9_i1 gave rise to vesicular structures, which colocalized with non-assembled MTs, as observed for endogenous septin 9 in JFH1-infected cells (Figure 3b). Interestingly, treating septin 9_i1-transfected cells with nocodazole (which depolymerizes microtubules) disrupted the association between septin 9_i1 and MTs, while septin 9_i1 formed notable stress fibers (Figure 4c), as previously been reported [59]. Conversely, the treatment of cells with Taxol, which stabilizes microtubule filaments, induced the formation of stronger septin 9_i1 filaments (Figure 4c), indicating that septin_i1 filament assembly is regulated by the microtubule network. We further used the septin 9_i1-transfected Huh7R cells transfected with PIAS1 siRNA (siPIAS1) to analyze the effect of PIAS1 knockdown on septin 9_i1 expression, revealed by immunoblotting using the V5 tag antibody. The results showed that PIAS1 knockdown decreased septin 9_i1 expression in the band detected at 75 kDa (Figure 4d). Together, these data indicate that PIAS1 also regulates septin 9_i1 filament formation.

### 2.4. PIAS1 Regulates Septin 9-Induced LD Accumulation

**Figure 5:**
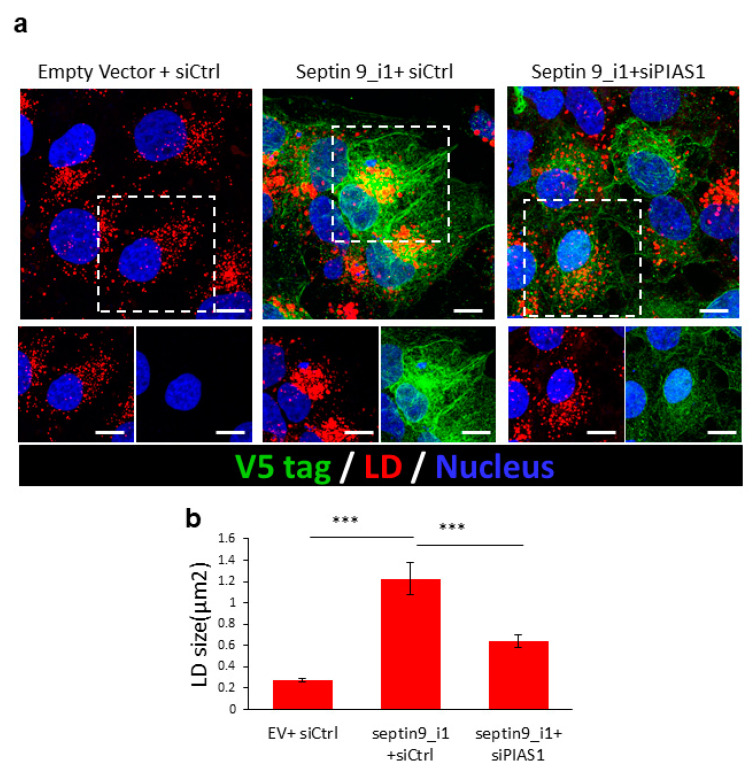
Knockdown of PIAS1 downregulates septin 9 and induces LD accumulation. (**a**) Huh7R cells were transfected with an empty vector or septin 9_i1 with or without PIAS1 siRNA, and stained for V5 (green) and LD (red). Scale bar, 10 µm. (**b**) The size of LDs was analyzed for at least 30 cells from the experiments described in (**a**). Data information: Bar graphs present the mean ± SEM. A Student’s *t*-test was used: *** *p* < 0.0001.

According to the effects of PIAS1 on septin 9 and MT filaments, we assessed the effect of PIAS1 depletion on the accumulation of LDs induced by septin 9_i1 (Figure 5a). Indeed, compared to cells transfected with an empty vector and control siRNA (Empty vector + siCtrl), the cells expressing septin 9_i1 (septin 9_i1 + siCtrl) displayed a higher content of LDs that formed a well-organized cluster surrounded by septin 9_v1 filaments. However, in cells co-transfected with septin 9_i1 and PIAS1 siRNA, the LDs were smaller than in septin 9_i1 cells and no clusters were observed. As expected, the formation of septin 9 filaments was also disrupted (Figure 5a,b). Here, again, these findings are very similar to those obtained with endogenous septin 9 in JFH1-infected cells (Figure 2) and reinforce the role of PIAS1 as a regulator of septin 9 filament organization, as well as its involvement in the accumulation and distribution of LDs.

## 3. Discussion

The progression of chronic HCV infection involves the development of fatty liver with the accumulation of LDs in hepatocytes. LDs play a crucial role in the HCV life cycle and, in most cases, interactions between HCV proteins (particularly the HCV core protein) and LDs are required for morphogenesis and the production of infectious HCV [55,60]. Although hundreds of cellular factors have been identified as being involved in the HCV life cycle [61], how they contribute to HCV pathogenesis remains unknown.

The involvement of the PIAS1 protein in HCV infection has already been demonstrated, and it is believed that the main function of PIAS1 during HCV infection is linked to its role as a negative regulator of STAT signaling. Thereby, the upregulation of PIAS1 expression alters that of the interferon (IFN) target genes that are important to cellular defenses against HCV [62,63]. During this study, we investigated the contribution of PIAS1 in HCV replication using a different approach. We first revealed the upregulation of PIAS1 expression following infection by HCV, which is in line with data that show an overexpression of this protein in liver biopsies from HCV patients [64]. In addition, we observed a colocalization of the HCV core and PIAS1 in the perinuclear region (Figure 1). Then, we demonstrated that PIAS1, in turn, controls the accumulation of LDs and the replication of HCV (Figure 2). Furthermore, our study revealed that PIAS1 modulates the redistribution of LDs and their association with the HCV core (Figure 2) and thus contributes to establishing a lipid environment appropriate for viral replication and production.

Another important finding was the identification of PIAS1 as a regulator of septin 9 assembly in filaments, as the knockdown of PIAS1 disrupted the filamentous structure of septin 9, which became vesicular (Figure 3). Herein, we also provided evidence regarding the interaction between septin 9 and PIAS1, since PIAS1 was found in septin 9_i1-immunoprecipitated proteins and, furthermore, the partial colocalization of septin_i1 and PIAS1 was observed by the immunofluorescence study of cells containing the HCV genomic replicon (Figure 4). In our previous study, we demonstrated the role of MTs and septin 9 in HCV infection [15]. Given the fact that septin filament assembly is tightly regulated by its SUMOylation [59], and, in yeast, this SUMOylation is mediated by yeast Siz proteins, the homologs of PIAS1 [48], further studies to reveal the role of PIAS1 in mammalian septin 9 SUMOylation and the pathogenesis of HCV are required. Overall, we suggest that in addition to its role as a STAT signaling regulator for HCV immune escape, PIAS1 is also hijacked by HCV to promote LD accumulation through the regulation of MT and septin 9, which are necessary for HCV replication.

To conclude, this work has provided fundamental data on the functional features of PIAS1 as a regulator of cytoskeleton elements such as MT and septin 9 and LD metabolism. This study also offers significant insights into the host cellular machineries exploited by HCV to enable its replication. Targeting PIAS1 may therefore enable new antiviral approaches to combat HCV infection.

## 4. Materials and Methods

### 4.1. Antibodies and Reagents

Anti-mouse β-actin came from Sigma, and anti-rabbit PIAS1, anti-V5 tag, and anti-mouse HCV core were obtained from Abcam. Anti-mouse Alexa Fluor 488 or 546 came from Invitrogen. Nuclei were stained with Hoechst from Invitrogen. Nocodazole and Taxol were obtained from Sigma. For immunoblotting and immunofluorescence, the antibody was diluted at a ratio of 1:500 and 1:100, respectively.

### 4.2. Cell Culture

Huh7.5, Huh7 cells harboring the HCV genome-length replicon, and Huh7.5-infected cells were cultured in Dulbecco’s Modified Eagle’s Medium (DMEM; Invitrogen, Paisley, UK) containing 4.5 g/L of glucose supplemented with 10% heat-inactivated fetal bovine serum, 1% nonessential amino acids (Invitrogen, Paisley, UK), and 1% penicillin/streptomycin (Invitrogen, Paisley, UK).

### 4.3. siRNAs and Cell Transfection

The endogenous expression of PIAS1 was silenced by transfection with a pool of specific siRNAs PIAS1 (si1: sc-36219 and si2: sc-36220; Santa Cruz, Dallas, TX, USA). Huh7.5 cells were grown in 12-well plates or on glass coverslips and transiently transfected with 75/100 pmol siRNA oligonucleotide of PIAS1 at 40–50% confluence using Lipofectamine RNAiMAX (Invitrogen, Paisley, UK) according to the manufacturer’s protocol. Control non-targeting siRNAs were used as a negative control (Genecust, Boynes, France). Depending on the experiments, cells were lysed in lysis buffer containing a cocktail of protease inhibitors (Roche, Meylan, France), fixed with 3.7% paraformaldehyde for immunofluorescence staining or used for the isolation of total RNA with RNAble solution (Eurobio, Les Ulis, France).

### 4.4. Immunoblotting

Cells were collected on ice, washed and lysed in 20 mM Tris, HCl, 100 mM NaCl, 1% Triton X100, and 10 mM EDTA at pH 7.4 containing a protease and phosphatase inhibitor cocktail (Roche Diagnostics). The proteins were separated on SDS-PAGE, blotted onto a PVDF membrane, and visualized using a chemiluminescence reagent (Amersham, Darmstadt, Germany).

### 4.5. Immunofluorescence Staining

Huh7.5 cells were grown on glass coverslips in 12-well plates, treated depending on the experiments, fixed with 3.7% paraformaldehyde, permeabilized, and saturated with PBS supplemented with 0.7% fish gelatin and 0.025% saponin. Primary antibodies were diluted with permeabilization solution and incubated with the cells. After several washes, staining was performed with fluorescent secondary antibodies and Hoechst. The lipophilic fluorescence dye LD 540 was used to stain the lipid droplets. For the nocodazole and Taxol treatments, the cells were cultured with 33 nM nocodazole or 500 nM Taxol containing medium 2 h prior to fixation.

### 4.6. JFH1/HCVcc Inoculation

Cells were infected with HCV at 37 °C for 3 h, and then the unbound viruses were removed by aspiration and by washing the cells three times with PBS. At 48 h post-infection, the infected cells were either lysed in lysis buffer (used for the isolation of total RNA) or fixed with 3.7% paraformaldehyde. The levels of HCV proteins in the HCV-infected cells were determined by Western blotting, while the levels of HCV RNA were quantified using the reverse transcription polymerase chain reaction (RT-PCR) method, and IFA staining was performed with fluorescent secondary antibodies.

### 4.7. Determination of Infectious HCV Titer

Serially diluted HCV was used to infect naive Huh-7.5 cells in 24-well plates. Three days post-infection, focus-forming units (FFUs) were determined by IFA staining using a specific monoclonal antibody. The infectious HCV titer was calculated from the average number of NS5A-positive FFU/mL in triplicate assays.

### 4.8. RNA Extraction and RT-PCR Analysis

Total RNA was isolated using an RNAble solution (Eurobio, Les Ulis, France) and intracellular levels of positive strand HCV RNA were quantified using a strand-specific qRT-PCR technique with a Light Cycler Fast Start DNA MasterPlus SYBR Green I mix (Roche, Mannheim, Germany) on a Light Cycler 480 Real-Time PCR System (Roche, Mannheim, Germany). The triplicate mean values were calculated according to the Ct quantification method using GAPDH gene transcription as the reference for normalization. Details on the methods using specific primers and probes are available on request. The average levels of HCV RNA compared to the positive control, obtained during triplicate experiments, are shown.

### 4.9. Image Acquisition and Analysis

Images were acquired with a Zeiss 510 LSM confocal microscope or Leica TCS SP5 (Leica Microsystems, available on April 2016). For colocalization analysis, images were treated by ImageJ software, and plugin “Intensity Correlation Analysis” was used to generate the Pearson’s correlation coefficient (Rr), which ranged from −1 (perfect exclusion) to +1 (perfect correlation).

To calculate the total intensity of the HCV core and LDs, images obtained by confocal microscopy were processed by cell ROI using the freehand selection tool, then the total intensity of the HCV core and LDs were measured by ImageJ analyses.

### 4.10. Statistical Analyses

Comparisons of mean values were conducted using unpaired Student’s *t*-tests. Statistical significance was determined at * *p* < 0.05, ** *p* < 0.001, and *** p < 0.0001.

## Data Availability

The data presented in this study are available in the main text, figures, tables.
